# Impaired *FHIT* expression characterizes serous ovarian carcinoma

**DOI:** 10.1054/bjoc.2001.1886

**Published:** 2001-07

**Authors:** K Ozaki, T Enomoto, K Yoshino, M Fujita, G S Buzard, K Kawano, M Yamasaki, Y Murata

**Affiliations:** Department of Obstetrics and Gynecology, Osaka University Faculty of Medicine, Osaka, Japan; Department of Pathology, Department of Obstetrics and Gynecology, Osaka Rosai Hospital, Osaka, Japan; Intramural Research Support Program, SAIC, Bldg 538, Rm 205D, P.O. Box B, NCI-Frederick, Frederick, MD, 21701, USA

**Keywords:** *FHIT* (*fragile histidine triad*) gene, ovarian tumour, serous carcinoma

## Abstract

The *FHIT* (*fragile histidine triad*) gene on chromosome 3p14.2 is a candidate tumour suppressor gene. To define the role of the *FHIT* gene in the development of ovarian cancer, we have examined 33 ovarian carcinomas, 2 borderline tumours and 10 benign adenomas for the presence of *FHIT* gene alterations. *FHIT* transcripts were analysed by RT-PCR and sequencing. Aberrant *FHIT* transcripts were observed in 5/33 carcinomas (15%) and in 1 of 2 borderline tumours. Loss of normal *FHIT* transcript was observed in 5/33 carcinomas (15%) but not in 2 borderline tumours or 10 benign adenomas. Allelic losses at D3S1300 and D3S4103, both located within intron 5 of *FHIT* were detected in 5/24 (21%) and 5/25 (20%) informative ovarian carcinomas, respectively. Expression of Fhit protein was analysed by immunohistochemistry in 44 carcinomas, 19 borderline tumours and 16 benign adenomas. Loss or significantly reduced expression of Fhit protein was observed in 6/44 (14%) ovarian carcinomas but not in any of 19 borderline tumours or 16 benign adenomas. The impaired Fhit protein expression was significantly correlated with the loss of normal *FHIT* transcription. Most notably, loss of normal *FHIT* transcript and impaired expression of Fhit protein occurred only in serous adenocarcinomas of grade 2 and 3 (5/15; 33% and 6/19; 32%, respectively). The present data suggest that inactivation of the *FHIT* gene by loss of expression is one of the important molecular events associated with the genesis of ovarian carcinoma, especially of high-grade serous carcinoma. © 2001 Cancer Research Campaign http://www.bjcancer.com


*British Journal of Cancer* (2001) **85**(2), 247–254
© 2001 Cancer Research Campaign
doi: 10.1054/ bjoc.2001.1886, available online at http://www.idealibrary.com on IDEAL®

### Impaired *FHIT* expression characterizes serous ovarian carcinoma

**K Ozaki[1], T Enomoto[1], K Yoshino[1], M Fujita[1], GS Buzard[3], K Kawano[2], M Yamasaki[2], and Y Murata[1]**

[1]Department of Obstetrics and Gynecology, Osaka University Faculty of Medicine, Osaka, Japan; [2]Department of Pathology, Department of Obstetrics and Gynecology, Osaka Rosai Hospital, Osaka, Japan; [3]Intramural Research Support Program, SAIC, Bldg 538, Rm 205D, P.O. Box B, NCI-Frederick, Frederick, MD, 21701 USA

**Summary** The *FHIT* (*fragile histidine triad*) gene on chromosome 3p14.2 is a candidate tumour suppressor gene. To define the role of the *FHIT* gene in the development of ovarian cancer, we have examined 33 ovarian carcinomas, 2 borderline tumours and 10 benign adenomas for the presence of *FHIT* gene alterations. *FHIT* transcripts were analysed by RT-PCR and sequencing. Aberrant *FHIT* transcripts were observed in 5/33 carcinomas (15%) and in 1 of 2 borderline tumours. Loss of normal *FHIT* transcript was observed in 5/33 carcinomas (15%) but not in 2 borderline tumours or 10 benign adenomas. Allelic losses at D3S1300 and D3S4103, both located within intron 5 of *FHIT*, were detected in 5/24 (21%) and 5/25 (20%) informative ovarian carcinomas, respectively. Expression of Fhit protein was analysed by immunohistochemistry in 44 carcinomas, 19 borderline tumours and 16 benign adenomas. Loss or significantly reduced expression of Fhit protein was observed in 6/44 (14%) ovarian carcinomas but not in any of 19 borderline tumours or 16 benign adenomas. The impaired Fhit protein expression was significantly correlated with the loss of normal *FHIT* transcription. Most notably, loss of normal *FHIT* transcript and impaired expression of Fhit protein occurred only in serous adenocarcinomas of grade 2 and 3 (5/15; 33% and 6/19; 32%, respectively). The present data suggest that inactivation of the *FHIT* gene by loss of expression is one of the important molecular events associated with the genesis of ovarian carcinoma, especially of high-grade serous carcinoma. © 2001 Cancer Research Campaign http://www.bjcancer.com

**Keywords**: *FHIT* (*fragile histidine triad*) gene; ovarian tumour; serous carcinoma

Among the human gynaecologic malignancies, the difficulty in early detection of ovarian cancer usually results in a poor patient prognosis. The incidence has been increasing, with over 5000 ovarian cancer-related deaths each year in Japan alone. Nearly 80% of ovarian malignant tumours are of the epithelial type, with multiple genetic changes involved in their genesis and progression, including the activation of proto-oncogenes and the inactivation of tumour suppressor genes (Stanbridge, 1990). Only now are the detailed molecular mechanisms of the development of ovarian tumours beginning to be elucidated.

In addition to the well-characterized tumour suppressor genes such as *RB, WT1*, and *p53*, several newer candidate tumour suppressor genes, including *Smad4* (Hahn et al, 1996) and *PTEN* (Li et al, 1997), have been identified through LOH analysis of human tumours. Although studies from ovarian cancers have shown that LOH occurs at some level on most chromosome arms, deletions were most frequently observed at 6p, 13q, 17p and 17q (Cliby et al, 1993). In several recent studies of ovarian carcinomas, LOH at 3p was detected in 15% (Buttitta et al, 1998), 31% (Lounis et al, 1998), and 52% of the tumours (Fullwood et al, 1999). The *FHIT* gene is located at 3p14.2, close to the chromosomal region shown to be susceptible to LOH and cytogentic abnormalities in ovarian carcinoma (Lounis et al, 1998). The *FHIT* (*fragile histidine triad*) gene is a probable tumour suppressor gene. Its 1.1-kb mRNA transcript is encoded by 10 exons distributed over an amazing 1 Mb of genomic DNA. *FHIT* encompasses the FRA3B fragile site and the breakpoint of the t(3:8) translocation associated with familial renal cell carcinoma (Ohta et al, 1996).

*Received 9 August 2000*
*Revised 2 January 2001*
*Accepted 11 January 2001*

*Correspondence to:* T Enomoto

In vitro studies show that the human Fhit protein has the properties of a diadenosine triphosphate hydrolase (Barnes et al, 1996). The tumour-suppressor activity of Fhit may be associated with an involvement of the Fhit/Ap3A complex in the cytokine signaling pathways which control cell proliferation (Kisselev et al, 1998). Overexpression of Fhit in cells with *FHIT* gene abnormalities or lacking Fhit protein expression significantly inhibits cell growth in human lung, head and neck, and colon carcinoma cells, and also causes an induction of tumour cell apoptosis and reduction of tumorigenicity (Ji et al, 1999; Sard et al, 1999; Morikawa et al, 2000) but not in normal bronchial epithelial cells (Ji et al, 1999) nor in 293 T human embryonic kidney carcinoma cells (Guo and Vishwanatha, 2000).

A high frequency of aberrant transcripts (30–80%) of *FHIT* has been reported in various human cancers. We previously reported that 43% of cervical carcinomas showed inactivation of both *FHIT* alleles (Yoshino et al, 1998), highlighting the importance of *FHIT* alteration in that tumour type. We extended our studies backwards to normal cervical epithelia and cervical intraepithelial neoplasias and found that inactivation of *FHIT* alterations also occur as a later event in the development of endometrial carcinoma (Ozaki et al, 2000). However, reports on the role of the *FHIT* gene in ovarian carcinoma are scarce. Mandai et al (1998) found concomitant expression of normal and abnormal *FHIT* transcripts in 39% of carcinomas and in 83% of borderline tumours and loss of normal *FHIT* transcript in 4 (8%) cases of carcinomas, all of which were poorly differentiated. On the other hand, Buttitta et al (1998) found an abnormal *FHIT* transcript in only 2 of 54 ovarian carcinomas. Therefore, reports on the role of *FHIT* in ovarian carcinomas are conflicting.

In the present study, we have analysed *FHIT* transcription using RT-PCR and sequencing. We have also performed Southern DNA analysis and LOH analysis to clarify the mechanism of the aberrant *FHIT* transcripts we found. Expression levels of Fhit protein were examined by immunohistochemical analysis.

### MATERIALS AND METHODS

### Tissue samples

For RT-PCR, 33 samples of ovarian carcinomas (15 serous, 5 mucinous, 5 endometrioid and 8 clear cell type), 2 ovarian borderline tumours (1 serous and 1 mucinous type) and 10 benign adenomas (5 serous and 5 mucinous type) were obtained from patients of the Osaka University Hospital. Surgically removed tissues were sampled for histological diagnosis, and the remainders were quick-frozen for subsequent extraction of DNA and RNA. Surgical staging was carried out according to the tumour-node-metastasis (TNM) classification system of malignant tumours defined by the International Union Against Cancer (Sobin and Wittekind, 1997). Patient age ranged from 16 to 80 years (mean: 49 years); surgical staging ranged from Ia to IV in these ovarian carcinomas. For immunohistochemistry, a new series was added to the above samples, making a total of 44 ovarian carcinomas, 19 borderline tumours, and 16 benign adenomas. These samples were obtained from the Osaka University Hospital and the Osaka Rosai Hospital.

### RNA extraction, reverse transcription-polymerase chain reaction (RT-PCR)

Total RNA was extracted from frozen tissues using the RNAzol B kit (Tel TEST, Friendswood, TX) according to the manufacturer's instructions. For cDNA synthesis, 1 μg of total RNA was reverse transcribed in a 30 μl volume of 1X first strand buffer, 10 mM DTT, 500 μM dNTPs and 0.3 μg $\mu l^{-1}$ of random hexamers. The samples were first denatured for 10 min at 70°C, annealed with random hexamers at 26°C for 10 min and then 10 units of MoMLV reverse transcriptase (Life Technologies, Gaithersburg, MD) were added. Samples were incubated at 37°C for 60 min. The reaction was stopped by inactivating the enzyme at 70°C for 15 min.

1 μl of this cDNA was used for the first round of PCR amplification in a volume of 25 μl containing 0.8 μM of primers 5U2 (5′-CATCCTGGAAGCTTTGAAGCTCA-3′) and 3D2 (5′-TCACTGGTTGAAGAATACAGG-3′), as published by Ohta et al (1996), 50 μM of each dNTP, 1X PCR buffer, and 1.25 units of ExTaq Polymerase (Takara Shuzo, Kyoto, Japan). The PCR consisted of an initial denaturation at 94°C for 2 min and 5 cycles of 30 s at 94°C, 3 min at 68°C, 5 cycles of 30 s at 94°C, 3 min at 66°C, 20 cycles of 20 s at 94°C, 3 min at 64°C, using a PCR Thermal Cycler (Perkin Elmer Cetus, Foster City, CA). The amplified product was diluted 10-fold with TE buffer (10 mM Tris-HC1, 1 mM EDTA), and then 1 μl of the diluted reaction product was subjected to a second round of PCR amplification using the nested primers 5U1 (5′-TCCGTAGTGCTATCTACATC-3′) and 3D1 (5′-CATGCTGATTCAGTTCCTCTTGG-3′), as published by Ohta et al (1996), under the conditions of an initial denaturation at 94°C for 2 min and 25 cycles of 30 s at 94°C, 30 s at 60°C, 1 min at 72°C. For control of RT-PCR, a 319-bp fragment of β-actin cDNA was also amplified using primers previously described (Fuqua et al, 1990). One cycle for the β-actin fragment consisted of 1 min each at 94°C, 50°C and 72°C. A total 30 cycles of PCR amplification was performed.

### cDNA sequencing

The PCR products were electrophoresed in a 1.5% agarose gel and visualized by ethidium bromide staining. DNA was purified from excised bands using a Gene Clean Kit (Bio 101, Vista, CA). The products were sequenced using a Dye-Terminator Cycle Sequencing Ready Reaction Kit (Perkin-Elmer) and run on an automatic DNA sequencer (Applied Biosystems Model 310 Genetic Analyzer).

### Detection of allelic loss within the *FHIT* gene

For analysis of allelic loss within the *FHIT* gene, DNA was extracted from the tumour and normal tissue and purified as previously described (Enomoto et al, 1991). Allelic loss was detected using amplification of D3S1300 and D3S4103 microsatellite markers, both of which are located within intron 5 of the *FHIT* gene. Primer sequences used in this study were obtained from the Genome Database, (5′-AGCTCACATTCTAGTCAGCCT-3′ [forward] and 5′-GCCAATTCCCCAGATG-3′ [reverse] for D3S1300, 5′-TTCTACTGCAATCCAGCCTGG-3′ [forward] and 5′-GCCTTGGGTAGATTTTATACCT-3′ [reverse] for D3S4103). The PCR amplification reaction mix (total 10 μl) contained genomic DNA (0.1 μg), dNTPs (50 μM), [$^{32}$P]-end-labelled reverse primer and unlabelled forward primer (1 μM each), $MgCl_2$ (1.5 mM), Tris, pH 8.3 (10 mM), KCl (50 mM), 0.1 U Taq polymerase (Takara Syuzo) and 0.01% gelatin. After initial denaturation at 98°C for 5 min, a total of 28 cycles of PCR were performed with a thermal profile of 1 min at 94°C, and 1 min at 60°C. The PCR products were analysed by electrophoresis through an 8% non-denaturing polyacrylamide gel.

### Southern blot analysis

For Southern blot analysis, 10 μg of genomic DNA was digested in a 20 μl reaction mix containing 1X restriction buffer and 80 units of restriction enzyme (*Hind* III; Takara, Japan). Digested DNA was electrophoresed on 0.8% agarose gel and transferred to a Hybond N membrane (Amersham Life Science, Inc, Arlington Heights, IL) according to the manufacturer's instructions. A 707-bp cDNA probe spanning exons 3 to 10 of the gene was generated by RT-PCR and was labelled with [α-$^{32}$P] dCTP using a Prime-It II Kit (Stratagene, La Jolla, CA). Hybridization was performed at 65°C in 7 ml of Quick Hyb (Stratagene, La, Jolla, CA) for 2 hours. Hybridized membranes were washed in 2 × SSC, 0.1% SDS for 20 min at room temperature, and in 0.2 × SSC, 0.1% SDS for 20 min at 65°C, and subjected to autoradiography.

### Immunohistochemical analysis of Fhit protein expression

Formalin-fixed, paraffin-embedded tissues were sectioned at 4 μm. Antigen enhancement was performed by heating the sections to 100°C in sodium citrate buffer (0.01 M, pH 6.0) for

 © 2001 Cancer Research Campaign

10 min. Slides were cooled for 20 min at room temperature, washed 3 times in phosphate buffer, treated for 10 min with 3% hydrogen peroxide in methanol to reduce endogenous peroxidase activity, and washed in water for 5 min. Slides were incubated with polyvalent and universal protein blocking agent for 20 min, then incubated with rabbit polyclonal anti-Fhit antiserum at 1: 500 dilution (Zymed Laboratories, Inc.) at 4°C for 16 h. Slides were again washed 2 times and incubated with polyvalent and universal blocking agent plus secondary antibody for 30 min. Avidin-biotin complex was added to the slides for 30 min, and antibody localization was detected by a 5-min incubation with 3,3′-diaminobenzidine. Slides were lightly counterstained with haematoxylin.

Immunostaining was scored on a 3-tiered scale for both intensity (grade 1: absent/weak, 2: moderate, 3: strong) and extent (grade 1: percentage of positive cells is <10%, grade 2: 10–50%, grade 3: >50%). The intensity and extent scores were then multiplied to give a composite score of 1–9 for each tumour (Greenspan et al, 1997). Composite scores from 1 to 3 were defined as marked reduction or absence of Fhit protein expression. Slides were scored independently by 2 investigators (KO and TE).

### Statistics

The significance of differences in the frequency with which mutations occurred in different histological grades, histological subtypes or surgical stages was estimated using Fisher's exact test.

### RESULTS

### RT-PCR and cDNA sequencing of *FHIT* gene

To study abnormal *FHIT* transcription in carcinogenesis of the ovary, 33 ovarian carcinomas, 2 borderline tumours and 10 benign adenomas were analysed for a 707-bp cDNA fragment encompassing exons 3 to 10 of the *FHIT* gene. *FHIT* cDNA was amplified by nested RT-PCR using tumour mRNA as template. A 319-bp fragment of β-actin was amplified from all the samples as an internal control (data not shown). We successfully amplified a normal-sized 707-bp *FHIT* fragment from all the borderline tumours and benign adenomas. On the other hand, 5 ovarian carcinomas (cases 18, 19, 22, 25 and 27) exhibited complete loss of the normal-sized 707-bp fragment; 2 of these (cases 22 and 27) expressed only an abnormal-sized transcript, and the remaining 3 expressed no *FHIT* transcripts at all. For comparison, examples of 7 cases with the normal-sized 707-bp fragment, a single case with the normal-sized 707-bp fragment and an abnormal-sized transcript, and a single case with only an abnormal-sized transcript, are shown in Figure 1.

Association of loss of the normal-sized 707-bp fragment with tumour histological subtypes and surgical stages was evaluated. Loss of the normal-sized 707-bp *FHIT* fragment was observed in 5 of 15 serous carcinomas but not in any of 18 non-serous carcinomas. Loss of the normal-sized 707-bp fragment was observed in 1 of 12, 1 of 6, 3 of 13 and none of 2 ovarian carcinomas of stage I, II, III and IV, respectively. Therefore, there was a significant association between loss of normal *FHIT* transcript and histological subtype ($P$ = 0.01, serous vs. non-serous), though no association was observed with surgical stages ($P$ = 0.70).

Abnormal *FHIT* transcripts were observed in 5 of 33 ovarian carcinomas (cases 20, 22, 24, 27, and 32) and in 1 of 2 borderline

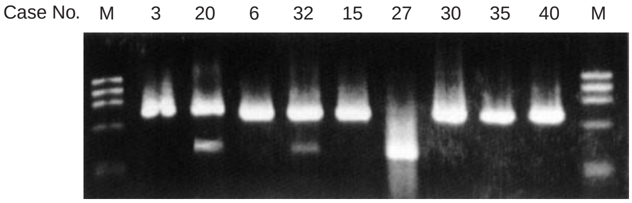

**Figure 1** RT-PCR analysis of *FHIT* mRNA in ovarian carcinomas. A 707-bp RT-PCR fragment encompassing exons 3 to 10 of the *FHIT* gene was amplified as described in 'Materials and Methods'. A 707-bp band of normal size was observed in all cases except for case 27. In cases 20 and 32, the amplified products consisted of 1 abnormal band and one normal-sized band. Case 27 showed one abnormal band with no normal-sized band. M: molecular size marker (Φ X174/*Hae* III digest)

tumours (case 12), but not in any of 10 adenomas. Of the 6 tumours, which expressed abnormal *FHIT* transcript, 4 cases (cases 12, 20, 24 and 32) also expressed a normal-sized 707-bp fragment; however, 2 cases (cases 22 and 27) did not (Table 1). The association of an abnormal *FHIT* transcript with different histological subtypes and surgical stages was evaluated. An abnormal *FHIT* transcript was observed in 4 of 16 serous tumours and 2 of 6 mucinous tumours, but not in any of 5 endometrioid carcinomas or 8 clear cell carcinomas. An abnormal *FHIT* transcript was detected in 1 of 12, 1 of 6, 3 of 13 and none of 2 carcinomas of surgical stage I, II, III and IV, respectively. Therefore, there was no apparent association between abnormal *FHIT* transcript and histological subtype ($P$ = 0.38, serous vs. non-serous) or surgical stages ($P$ = 0.70).

All PCR products were excised from an agarose gel and subsequently sequenced. In case 20, the abnormal transcript corresponding to the absence of exons 3 through 6 resulted from a fusion of exons 2 and 7. In case 22, 2 types of aberrant transcripts were revealed, loss of exons 4 to 6 and loss of exons 5 and 6, creating a junction between exons 3 and 7, and exons 4 and 7, respectively. In cases 24 and 32, the abnormal transcripts showed a lack of exons 4 to 6, corresponding to the fusion of exons 3 and 7 (Figure 2B). In cases 12 and 27, the abnormal transcripts showed lack of exons 4 to 8 (Figure 2A), corresponding to the fusion of exons 3 and 9. In all the abnormal transcripts detected the deletions occurred at the exon-intron boundary of a regular splice site. Sequencing analysis of normal-sized transcripts showed no deletion or insertion in any of the cases.

### Detection of allelic loss within the *FHIT* gene

Allelic loss within intron 5 of the *FHIT* gene was detected by amplification of the D3S1300 and D3S4103 microsatellite markers (Figure 3). Allelic loss at the D3S1300 locus was found in 5/24 (21%) informative cases of carcinoma, 1/2 (50%) informative cases of borderline tumours and 0/10 (0%) informative cases of adenomas. Similarly, allelic loss at the D3S4103 microsatellite marker was found in 5/25 (20%) informative cases of carcinomas and 0/10 informative cases of adenomas. Among cases that showed abnormal *FHIT* transcripts, 5 of 6 (83%) informative cases at the D3S1300 locus and 2 of 3 (67%) informative cases at the D3S4103 locus showed allelic loss. Among cases that showed loss of normal-sized *FHIT* fragments, 2 of 5 (40%) informative cases at the D3S1300 locus and 1 of 2 (50%) informative cases at the D3S4103 locus showed allelic loss.

© 2001 Cancer Research Campaign 

**Table 1** *Fhit* alterations in ovarian tumours

| No. | Age | Histology | Stage | *Fhit* Protein Expression | | | *Fhit* mRNA | LOH | |
|---|---|---|---|---|---|---|---|---|---|
| | | | | I | E | C | | D3S1300 | D3S4103 |
| **Adenoma** | | | | | | | | | |
| 01 | 33 | serous adenoma | | 3 | 3 | 9 | N | – | – |
| 02 | 37 | serous adenoma | | 3 | 3 | 9 | N | – | – |
| 03 | 54 | serous adenoma | | 3 | 3 | 9 | N | – | – |
| 04 | 28 | serous adenoma | | N.D. | | | N | – | – |
| 05 | 61 | serous adenoma | | N.D. | | | N | – | – |
| 06 | 44 | mucinous adenoma | | 3 | 3 | 9 | N | – | – |
| 07 | 52 | mucinous adenoma | | 2 | 3 | 6 | N | – | – |
| 08 | 63 | mucinous adenoma | | N.D. | | | N | – | N.I. |
| 09 | 42 | mucinous adenoma | | N.D. | | | N | – | – |
| 10 | 28 | mucinous adenoma | | N.D. | | | N | – | – |
| **<Borderline tumour>** | | | | | | | | | |
| 11 | 51 | serous | Ic | 3 | 3 | 9 | N | – | N.D. |
| 12 | 23 | mucinous | Ia | 2 | 2 | 4 | N/A Del. Exon 4–8 | + | N.I. |
| **<Carcinoma >** | | | | | | | | | |
| 13 | 46 | serous G1 | Ic | 3 | 3 | 9 | N | N.I. | – |
| 14 | 53 | serous G1 | IIb | N.D. | | | N | – | – |
| 15 | 42 | serous G1 | IIIc | N.D. | | | N | – | – |
| 16 | 50 | serous G1 | IIIc | 3 | 2 | 6 | N | – | – |
| 17 | 43 | serous G1 | IV | 3 | 3 | 9 | N | N.D. | + |
| 18 | 55 | serous G2 | IIb | 1 | 2 | 2 | N.E. | – | – |
| 19 | 60 | serous G2 | IIIc | 1 | 1 | 1 | N.E. | – | – |
| 20 | 37 | serous G2 | IIIc | 2 | 3 | 6 | N/A Del. Exon 3–6 | + | + |
| 21 | 60 | serous G2 | IV | N.D. | | | N | – | N.D. |
| 22 | 55 | serous G3 | Ic | 1 | 1 | 1 | A1/A2 Del. Exon 4–6 / Del. Exon 5–6 | + | N.I. |
| 23 | 50 | serous G3 | IIc | 2 | 3 | 6 | N | – | – |
| 24 | 46 | serous G3 | IIc | 2 | 2 | 4 | N/A Del. Exon 4–6 | – | – |
| 25 | 45 | serous G3 | IIIc | 1 | 1 | 1 | N.E. | – | – |
| 26 | 63 | serous G3 | IIIc | 3 | 3 | 9 | N | – | – |
| 27 | 70 | serous G3 | IIIc | 1 | 2 | 2 | A Del. Exon 4–8 | + | + |
| 28 | 80 | mucinous G1 | Ia | 3 | 3 | 9 | N | – | – |
| 29 | 16 | mucinous G1 | Ia | 3 | 3 | 9 | N | – | N.D. |
| 30 | 55 | mucinous G1 | Ic | N.D. | | | N | – | N.I. |
| 31 | 45 | mucinous G1 | Ic | 3 | 3 | 9 | N | N.D. | + |
| 32 | 31 | mucinous G1 | IIIc | 3 | 3 | 9 | N/A Del. Exon 4–6 | + | N.I. |
| 33 | 39 | endometrioid G1 | IIIc | N.D. | | | N | – | – |
| 34 | 62 | endometrioid G1 | IIIc | 3 | 3 | 9 | N | – | – |
| 35 | 58 | endometrioid G2 | Ia | 3 | 3 | 9 | N | N.D. | + |
| 36 | 43 | endometrioid G2 | Ib | 3 | 3 | 9 | N | + | N.D. |
| 37 | 49 | endometrioid G2 | IIIc | 3 | 3 | 9 | N | N.D. | – |
| 38 | 59 | clear cell | Ia | 3 | 3 | 9 | N | – | – |
| 39 | 72 | clear cell | Ic | 3 | 3 | 9 | N | – | – |
| 40 | 31 | clear cell | Ic | 3 | 3 | 9 | N | – | – |
| 41 | 48 | clear cell | Ic | 3 | 3 | 9 | N | N.I. | – |
| 42 | 59 | clear cell | IIc | 3 | 3 | 9 | N | – | N.D. |
| 43 | 47 | clear cell | IIb | 3 | 3 | 9 | N | N.D. | – |
| 44 | 56 | clear cell | IIIc | 3 | 2 | 6 | N | N.D. | – |
| 45 | 58 | clear cell | IIIc | 3 | 3 | 9 | N | N.I. | N.D. |

N.D.: not done, N: normal-sized band, A: aberrant-sized band. N.E.: not expressed, Del: deletion, N.I.: not informative, +: LOH positive, –: no LOH. I: Intensity of immunohistochemical staining: 1, absent/weak; 2, moderate; 3, strong. E: Extent of immunohistochemical staining (percentage of positive cells): 1, <10%; 2, 10–50%; 3, >50%. C: Composite score: Intensity times Extent.

### Southern blot analysis

We carried out Southern blot hybridization analysis of genomic DNA digested with *Hind* III to clarify whether the aberrant *FHIT* transcripts are associated with DNA deletion or rearrangement. In all 3 cases (cases 12, 22 and 27) which showed both an aberrant *FHIT* transcript and LOH within intron 5, no structural abnormality was observed compared to control normal tissue DNA (Figure 4). This may indicate that the abnormal transcripts in these cases are not associated with gross DNA alterations such as deletion or rearrangement, but rather from altered splicing.

### Immunohistochemical analysis of Fhit protein expression

Expression levels of Fhit protein were analysed by immunohistochemistry (Figure 5) in 44 ovarian carcinomas (19 serous

 *© 2001 Cancer Research Campaign*

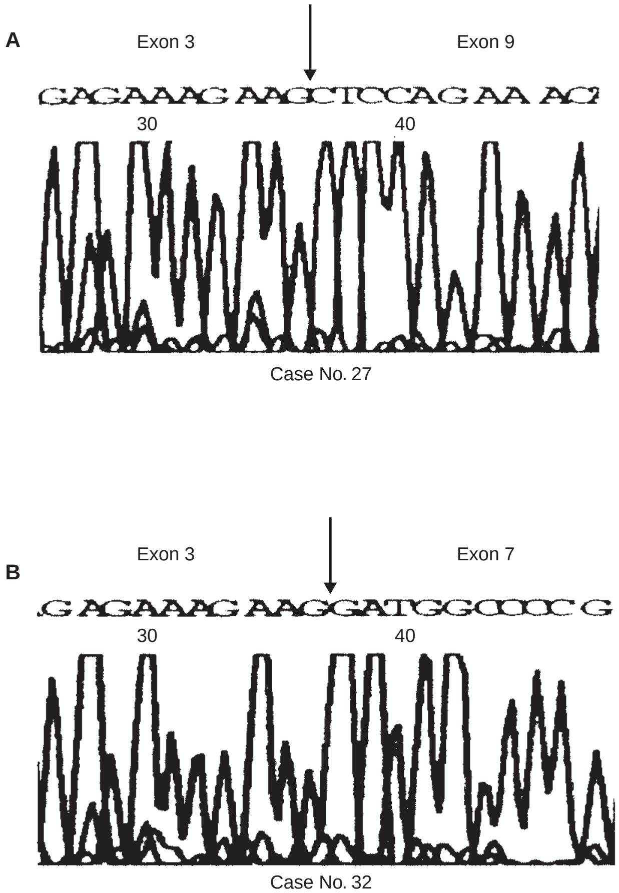

**Figure 2** Sequence of the abnormal-sized transcripts observed in ovarian carcinomas. Arrows indicate the junction between exon 3 and 9 in case 27 (A) and exon 3 and 7 in case 32 (B)

adenocarcinomas, 8 mucinous adenocarcinomas, 8 endometrioid adenocarcinomas and 9 clear cell carcinomas), 19 borderline tumours (8 serous, 11 mucinous) and 16 benign adenomas (8 serous adenomas, 8 mucinous adenomas). All borderline tumours

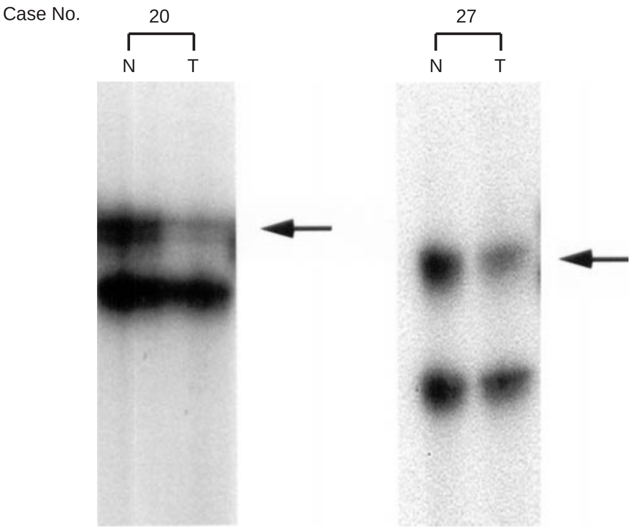

**Figure 3** LOH analysis of the D3S1300 and D3S4103 within the *FHIT* gene. Arrows indicate the allelic losses in cases 20 (D3S1300) and 27 (D3S4103). T: tumour, N: matched normal tissue

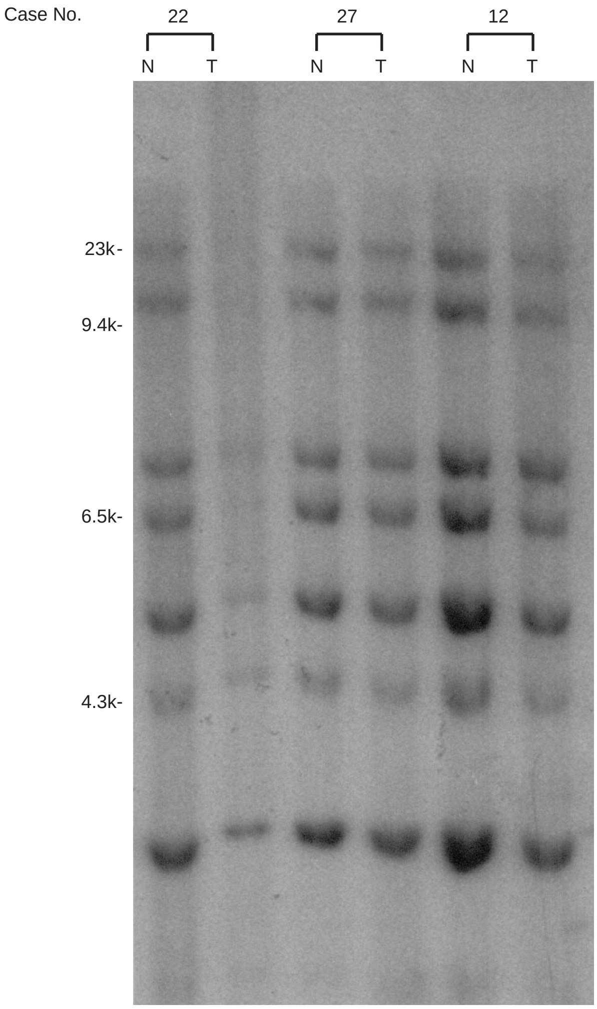

**Figure 4** Southern blot analysis of the *FHIT* gene in ovarian carcinomas. Tumour (T) and matched normal DNA (N) from cases 12, 22 and 27 were digested by *Hind* III and hybridized with a 707-bp *FHIT* cDNA probe and benign adenomas showed positive staining (composite score = 6–9) in the cytoplasm of epithelial components, whereas stromal tissues were negative for staining. In carcinomas, impaired expression of Fhit protein (composite score equal to or less than 3) was

**Table 2** Impaired *Fhit* expressions in ovarian tumours

| Histology | Composite score: 3 ⩾ | Loss of normal *Fhit* transcript |
|---|---|---|
| Adenoma | | |
| Serous | 0/8 (0%) | 0/5 (0%) |
| Mucinous | 0/8 (0%) | 0/5 (0%) |
| Borderline tumour | | |
| Serous | 0/8 (0%) | 0/1 (0%) |
| Mucinous | 0/11 (0%) | 0/1 (0%) |
| Carcinoma | | |
| Serous | 6/19 (32%)* | 5/15 (33%)** |
| Mucinous | 0/8 (0%) | 0/5 (0%) |
| Clear cell | 0/9 (0%) | 0/8 (0%) |
| Endometrioid | 0/8 (0%) | 0/5 (0%) |

*Impaired *fhit* expression of serous vs non-serous $P = 0.004$.
**Loss of *fhit* mRNA expression of serous vs non-serous $P = 0.01$.

*© 2001 Cancer Research Campaign*

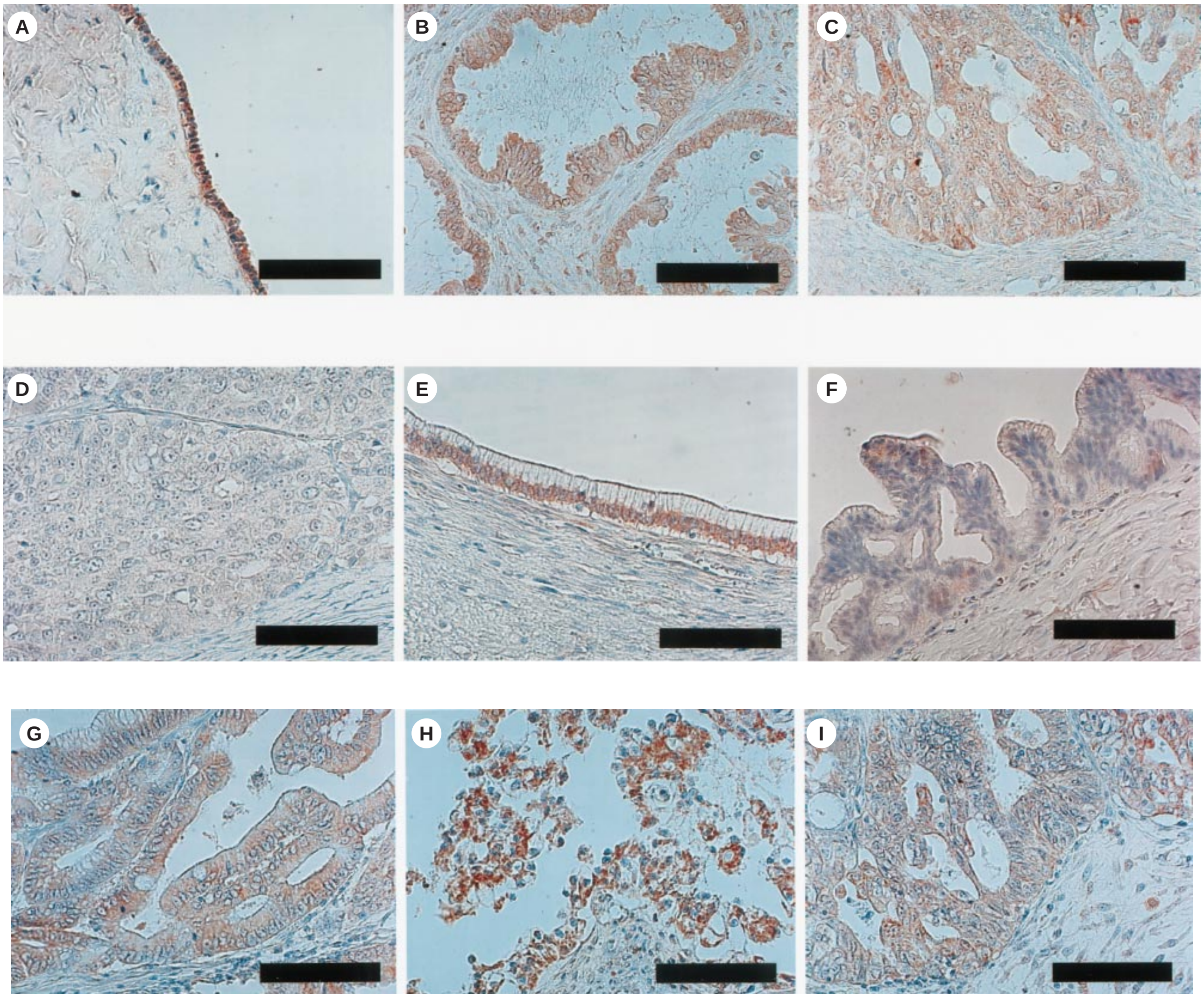

**Figure 5** Immunohistochemical detection of the Fhit protein in serous type tumours (**A**–**D**), mucinous type tumours (**E**–**G**), clear cell carcinoma (H), and endometrioid carcinoma (**I**). A case of serous adenoma (case 1) (**A**), a case of G1 serous adenocarcinoma (case 13) (**B**) and a case of G2 serous adenocarcinoma (**C**) showed strong positive staining (composite score = 9), whereas normal stromal tissue showed negative staining. G3 serous adenocarcinoma (case 27) (**D**) showed a weak staining (composite score = 2). A case of mucinous adenoma (case 6) (**E**) and a case of mucinous adenocarcinoma (case 28) (**G**) showed positive staining (composite score = 9), but one borderline mucinous tumour (case 12) (**F**) stained partially (composite score = 4). A case of clear cell carcinoma (case 40) (**H**) and a case of endometrioid adenocarcinoma (case 35) (**I**) showed positive staining (composite score = 9). Scale bar: 100 µm detected in 6 of 19 (32%) serous adenocarcinomas, but not in any of 25 non-serous carcinomas ($P$ = 0.004, serous vs. non-serous) (Table 2).

Association of impaired expression of the *FHIT* gene product and aberrant *FHIT* mRNA expression was evaluated. All 3 cases lacking *FHIT* transcripts (cases 18, 19 and 25) also failed to express Fhit protein. In addition, both cases that expressed only aberrant-sized *FHIT* transcripts (cases 22 and 27) expressed no Fhit protein. In contrast, the 4 cases that expressed an aberrant-sized *FHIT* transcript together with a normal-sized *FHIT* transcript (cases 12, 20, 24 and 32) each expressed some detectable Fhit protein. Therefore, the impaired expression of the Fhit protein was significantly correlated with the loss of normal *FHIT* transcription ($P$ = 0.01). In serous carcinoma, the correlation of impaired Fhit expression with histological grades did not quite achieve the level of statistical significance ($P$ = 0.26, Fisher's exact test; G1 vs. G2 + G3 = 0/6 vs. 2/6 + 4/9).

### DISCUSSION

It is believed that at least some fractions of ovarian carcinoma arise from the progressive transformation of ovarian surface epithelium through either benign tumours or borderline tumours. Mutations involving different proto-oncogenes and tumour suppressor genes accumulate during this process of malignant transformation. There is growing evidence that specific genes are associated with each histological subtype of this tumour. For example, early and frequent activation of the K-*ras* proto-oncogene is characteristic of the mucinous tumours and distinguishes them from other histological types (Enomoto et al, 1991). Also, alteration of the *p53* and DCC tumour suppressor genes is more characteristic of serous tumours than non-serous tumours (Fujita et al, 1994; Enomoto et al, 1995). The delayed timing of the inactivation of these genes suggests they may be

 © 2001 Cancer Research Campaign directly associated with malignant transformation (Fujita et al, 1994; Saegusa et al, 2000). Microsatellite instability and alteration of the *TGF beta-type II receptor* and the *PTEN* genes are more characteristic of endometrioid adenocarcinomas of the ovary (Lynch et al, 1998; Obata et al, 1998). No genetic alterations occurring specifically in clear cell carcinoma have been reported.

The present observations indicate that impaired Fhit protein expression occurred frequently in serous carcinomas but not in other histological sub-types (Table 2), and that impaired Fhit protein expression occurs in serous tumours of higher histological grade (grade 2 or 3), but not in grade 1 serous carcinomas, or serous tumours of low malignant potential. This suggests that inactivation of *FHIT* gene by loss of expression may play a major role in malignant transformation of the serous tumours but may not be as involved in tumours of other histological subtypes.

We show that impaired Fhit protein expression is more significantly correlated with the loss of the normal-sized *FHIT* transcript than with the presence of abnormal-sized *FHIT* transcripts. Although the 6 tumours with abnormal-sized transcripts lacked a variable number of exons, it is noteworthy that each had deletions that included exon 5, where the authentic translation-initiation methionine codon is located. The abnormal-sized transcripts are thus unlikely to encode full length normally functioning Fhit proteins.

Aberrant transcripts of the *FHIT* gene have been reported in a variety of human cancers, including lung, colon, oesophageal, breast, head and neck, cervical and endometrial carcinomas. These aberrant transcripts are not always associated with the presence of genomic deletions within the *FHIT* locus. Mao et al (1996) reported LOH of the *FHIT* locus in 13 of 16 head and neck cancer cell lines. They found that only 5 of the 13 cell lines with LOH had aberrant *FHIT* transcripts; the remaining 8 cases of LOH had normal *FHIT* transcription. In 2 of the 3 cell lines without LOH there was either an aberrant transcript or no transcript. Our analysis of the Fhit locus of 3 ovarian carcinomas gave results supporting and extending the findings of Mao et al (1996), that aberrant *FHIT* transcription is not necessarily associated with DNA rearrangement.

It should be noted that, curiously, aberrant *FHIT* transcripts have been frequently reported in many apparently histologically normal tissues, such as peripheral-blood lymphocytes, skeletal muscle and liver (Panagopoulos et al, 1997). We ourselves have previously reported that 10 of 40 histologically normal cervical tissues expressed aberrant *FHIT* transcripts together with normal *FHIT* transcript. Loss of the normal-sized *FHIT* mRNA transcript, and the corresponding loss of expression of Fhit protein, were observed in cervical and endometrial carcinomas, but were not found in normal cervical and endometrial tissue (Ozaki et al, 2000; Yoshino et al, 2000).

In the present study, loss of Fhit protein expression significantly correlated with the complete loss of *FHIT* transcripts but not with the presence of aberrant-sized *FHIT* transcripts. These observations suggest that loss of *FHIT* transcripts and the logically corresponding loss of Fhit protein expression is the major mechanism of inactivation of the *FHIT* gene in gynaecologic malignancies. In conclusion, a considerable contribution of the loss of Fhit protein expression in the development of specifically serous ovarian cancer was found in the present study.

### ACKNOWLEDGEMENTS

This project was funded in whole or in part with federal funds from the National Cancer Institute, National Institutes of Health, under contract NO1-CO-56000. The content of this publication does not necessarily reflect the views or policies of the Department of Health and Human Services, nor does mention of trade names, commercial products, or organizations imply endorsement by the U.S. Government.

### REFERENCES

Barnes LD, Garrison PN, Siprashvili Z, Guranowski A, Robinson AK, Ingram SW, Croce CM, Ohta M and Huebner K (1996) *FHIT*, a putative tumor suppressor in humans, is a dinucleoside 5′, 5′′′-P1, P3-triphosphate hydrolase. *Biochemistry* **35**: 11529–11535

Buttitta F, Marchetti A, Radi O, Pellegrini S, Gadducci A, Genazzani AR and Bevilacqua G (1998) Evaluation of *FHIT* gene alterations in ovarian cancer. *Br J Cancer* **77**: 1048–1051

Cliby W, Ritland S, Hartmann L, Dodson M, Halling KC, Keeney G, Podratz KC and Jenkins RB (1993) Human epithelial ovarian cancer allelotype. *Cancer Res* **15**: 2393–2398

Enomoto T, Inoue M, Perantoni AO, Buzard GS, Miki H, Tanizawa O and Rice JM (1991a) K-*ras* activation in premalignant and malignant epithelial lesions of the human uterus. *Cancer Res* **51**: 5308–5314

Enomoto T, Weghorst CM, Inoue M, Tanizawa O and Rice JM (1991b) K-*ras* activation occurs frequently in mucinous adenocarcinomas and rarely in other common epithelial tumors of the human ovary. *Am J Pathol* **139**: 777–785

Enomoto T, Fujita M, Cheng C, Ozaki M, Inoue M and Nomura T (1995) Loss of expression and loss of heterozygosity in the *DCC* gene in neoplasms of the human female reproductive tract. *Br J Cancer* **71**: 462–467

Fujita M, Enomoto T, Inoue M, Rice JM, Nomura T and Tanizawa O (1994) Alterations of the *p53* tumor suppressor gene occurs independently of K-*ras* activation and more frequently in serous adenocarcinomas than in other common epithelial tumors of the human ovary. *Jpn J Cancer Res* **85**: 1247–1256

Fullwood P, Marchini S, Radar JS, Martinez A, Macartney D, Broggini M, Morelli C, Barbanti-Brodano G, Maher ER and Latif F (1999) Detailed genetic and physical mapping of tumor suppressor loci on chromosome 3p in ovarian cancer. *Cancer Res* **59**: 4662–4667

Fuqua SA, Falette NF and McGuire WL (1990) Sensitive detection of estrogen receptor RNA by polymerase chain reaction assay. *J Natl Cancer Inst* **82**: 858–861

Greenspan DL, Connolly DC, Wu R, Lei RY, Vogelstein JT, Kim YT, Mok JE, Munoz N, Bosch FX, Shah K and Cho KR (1997) Loss of *FHIT* expression in cervical carcinoma cell lines and primary tumors. *Cancer Res* **57**: 4692–4698

Guo Z and Vishwanatha JK (2000) Effect of regulated expression of the *fragile histidine triad* gene on cell cycle and proliferation. *Mol Cell Biochem* **204**: 83–88

Hahn SA, Schutte M, Hoque AT, Moskaluk CA, da Costa LT, Rozenblum E, Weinstein CL, Fischer A, Yeo CJ, Hruban RH and Kern SE (1996) *DPC4*, a candidate tumor suppressor gene at human chromosome 18q21.1. *Science* **271**: 350–353

Ji L, Fang B, Yen N, Fong K, Minna JD and Roth JA (1999) Induction of apoptosis and inhibition of tumorigenicity and tumor growth by adenovirus vector-mediated fragile histidine triad (*FHIT*) gene over-expression. *Cancer Res* **59**: 3333–3339

Kisselev LL, Justesen J, Wolfson AD and Frolova LY (1998) Diadenosine oligophosphates (Ap(n)A), a novel class of signaling molecules? *FEBS Lett* **427**: 157–163

Li J, Yen C, Liaw D, Podsypanina K, Bose S, Wang SI, Puc J, Miliaresis C, Rodgers L, McCombie R, Bigner SH, Giovanella BC, Ittmann M, Tycko B, Hibshoosh H, Wigler MH and Parsons R (1997) *PTEN*, a putative protein tyrosine phosphatase gene mutated in human brain, breast, and prostate cancer. *Science* **275**: 1943–1947

Lounis H, Mes-Masson AM, Dion F, Bradley WE, Seymour RJ, Provencher D and Tonin PN (1998) Mapping of chromosome 3p deletions in human epithelial ovarian tumors. *Oncogene* **17**: 2359–2365

Lynch MA, Nakashima R, Song H, DeGroff V, Wang D, Enomoto T and Weghorst CM (1998) Mutational analysis of the *transforming growth factor beta-receptor type II* in human ovarian carcinoma. *Cancer Res* **58**: 4227–4232

© 2001 Cancer Research Campaign 

Mandai M, Konishi I, Kuroda H, Nanbu K, Matushita K, Yura Y, Hamid AA and Mori T (1998) Expression of abnormal transcripts of the *FHIT* (Fragile Histidine Triad) gene in ovarian carcinoma. *Eur J Cancer* **34**: 745–749

Mao L, Fan YH, Lotan R and Hong WK (1996) Frequent abnormalities of *FHIT*, a candidate tumor suppressor gene, in head and neck cancer cell lines. *Cancer Res* **56**: 5128–5131

Morikawa H, Nakagawa Y, Hashimoto K, Niki M, Egashira Y, Hirata I, Katsu K, Akao Y (2000) Frequent altered expression of fragile histidine triad protein in human colorectal adenomas. *Biochem Biophys Res Commun* **278**: 201–210

Obata K, Morland SJ, Watson RH, Hitchcock A, Chenevix Thomas EJ and Campbell IG (1998) Frequent *PTEN/MMAC* mutations in endometrioid but not serous or mucinous epithelial ovarian tumors. *Cancer Res* **58**: 2095–2097

Ohta M, Inoue H, Cotticelli MG, Kastury K, Baffa R, Palazzo J, Siprashvili Z, Mori M, McCue P, Druck T, Croce CM and Huebner K (1996) The *FHIT* gene, spanning the chromosome 3p14.2 fragile site and renal carcinoma-associated t(3;8) breakpoint, is abnormal in digestive tract cancers. *Cell* **84**: 587–597

Ozaki K, Enomoto T, Yoshino K, Sun H, Nakamura T, Kuragaki C, Fujita M, Kurachi H and Murata Y (2000) *FHIT* alterations in endometrial carcinoma and hyperplasia. *Int J Cancer* **85**: 306–312

Panagopoulos I, Thelin S, Mertens F, Mitelman F and Aman P (1997) Variable *FHIT* transcripts in non-neoplastic tissues. *Genes Chromosomes Cancer* **19**: 215–219

Saegusa M, Machida D and Okayasu I (2000) Loss of *DCC* gene expression during ovarian tumorigenesis: relation to tumour differentiation and progression. *Br J Cancer* **82**: 571–578

Sard L, Accornero P, Tornielli S, Delia D, Bunone G, Campiglio M, Colombo MP, Gramegna M, Croce CM, Pierotti MA and Sozzi G (1999) The tumor-suppressor gene *FHIT* is involved in the regulation of apoptosis and in cell cycle control. *Proc Natl Acad Sci USA* **96**: 8489–8492

Sobin LH and Wittekind C (1997) *TNM: classification of malignant tumours* (5th ed.). Wiley-Liss, New York, pp. 147–151

Stanbridge EJ (1990) Human tumor-suppressor genes. *Annu Rev Genet* **24**: 615–657

Yoshino K, Enomoto T, Nakamura T, Nakashima R, Wada H, Saitoh J, Noda K and Murata Y (1998) Aberrant *FHIT* transcripts in squamous cell carcinoma of the uterine cervix. *Int J Cancer* **76**: 176–181

Yoshino K, Enomoto T, Ozaki K, Nakashima R, Wada H, Sun H, Saitoh J, Noda K and Murata Y (2000) *FHIT* alterations in cancerous and non-cancerous cervical epithelium. *Int J Cancer* **85**: 6–13

© 2001 Cancer Research Campaign
